# Effects of Few-Layer Graphene on the Sexual Reproduction of Seed Plants: An In Vivo Study with *Cucurbita pepo* L.

**DOI:** 10.3390/nano10091877

**Published:** 2020-09-19

**Authors:** Davide Zanelli, Fabio Candotto Carniel, Marina Garrido, Lorenzo Fortuna, Massimo Nepi, Giampiero Cai, Cecilia Del Casino, Ester Vázquez, Maurizio Prato, Mauro Tretiach

**Affiliations:** 1 Department of Life Sciences, University of Trieste, via L. Giorgieri 10, I-34127 Trieste, Italy; davide.zanelli@phd.units.it (D.Z.); tretiach@units.it (M.T.); 2 Department of Chemical and Pharmaceutical Science, University of Trieste, via L. Giorgieri 1, I-34127 Trieste, Italy; mgarrido@units.it (M.G.); lfortuna@units.it (L.F.); prato@units.it (M.P.); 3 Department of Life Sciences, University of Siena, via P. A. Mattioli 4, I-53100 Siena, Italy; massimo.nepi@unisi.it (M.N.); giampiero.cai@unisi.it (G.C.); cecilia.delcasino@unisi.it (C.D.C.); 4 Department of Organic Chemistry, Faculty of Chemical Science and Technology, University of Castilla-La Mancha, Av. Camilo José Cela, s/n, E-13005 Ciudad Real, Spain; ester.vazquez@uclm.es; 5 Instituto Regional de Investigación Científica Aplicada (IRICA), Universidad de Castilla-La Mancha, E-13071 Ciudad Real, Spain; 6 Center for Cooperative Research in Biomaterials (CIC biomaGUNE), Basque Research and Technology Alliance (BRTA), Paseo de Miramón 182, 20014 Donostia San Sebastián, Spain; 7 Basque Foundation for Science, Ikerbasque, 48013 Bilbao, Spain

**Keywords:** stigmatic surface, pollen germination, nanomaterials, flowers, particulate matter

## Abstract

Products containing graphene-related materials (GRMs) are becoming quite common, raising concerns for environmental safety. GRMs have varying effects on plants, but their impact on the sexual reproduction process is largely unknown. In this study, the effects of few-layer graphene (FLG) and a similarly layered phyllosilicate, muscovite mica (MICA), were tested in vivo on the reproductive structures, i.e., pollen and stigma, of *Cucurbita pepo* L. ssp. *pepo* ‘*greyzini*’ (summer squash, zucchini). Pollen was exposed to FLG or MICA, after careful physical-chemical characterization, at concentrations of 0.5 and 2 mg of nanomaterial (NM) per g of pollen for up to six hours. Following this, pollen viability was tested. Stigmas were exposed to FLG or MICA for three hours and then analyzed by environmental scanning electron microscopy to verify possible alterations to their surface. Stigmas were then hand-pollinated to verify the effects of the two NMs on pollen adhesion and in vivo pollen germination. FLG and MICA altered neither pollen viability nor the stigmatic surface. However, both NMs equivalently decreased pollen adhesion and in vivo germination compared with untreated stigmas. These effects deserve further attention as they could impact on production of fruits and seeds. Importantly, it was shown that FLG is as safe as a naturally occurring nanomaterial.

## 1. Introduction

Graphene is a nanomaterial (NM) made of carbon atoms arranged in a 2D crystal structure with remarkable physicochemical properties [1]. These make graphene suitable for numerous applications ranging from optoelectronics, to medical, to material engineering [2,3,4]. Nowadays, graphene-containing materials are used for applications and objects such as sports equipment, earbuds, and mobile covers and pads (for a list, visit www.graphene-info.com).

Despite the great benefits graphene brings to our lives, its uncontrolled, fast diffusion might also cause some drawbacks. Even if graphene and graphene-related materials (GRMs) are present in small amounts in the aforementioned objects, it is expected that the materials will get involuntarily released into the environment during their life cycle. At the same time, more and more applications involving the direct release of GRMs into the environment are under development, such as drugs enhancers and carriers, pesticides and fertilizers for crops [5,6], or sand improvers for soil remediation [7]. Furthermore, these applications raise concerns of a possible negative impact on ecosystems. Indeed, GRM particles of micro- and nanometric size are extremely lightweight and, thus, could easily be aerodispersed for long distances, as documented for carbon black in fine and ultrafine particulate matter (PM) [8].

The aerial organs of seed plants, i.e., leaves, stems, and flowers, are natural traps for PM [9,10] and may also intercept airborne GRMs. So far, widely varying effects of GRMs on seed plants have been reported, from positive to negative, possibly owing to different experimental conditions (materials, concentrations, exposure time, protocols, etc.) and/or species tested [11,12].

Recently, in vitro testing [13] on the pollen of *Corylus avellana* (common hazel) and *Nicotiana tabacum* (tobacco plant) showed that few-layer graphene (FLG) and graphene oxide (GO) can impair pollen germination and, in the case of GO, pollen tube growth [14,15]. In regard to GO, this effect was mainly caused by the acidic property of the material, which significantly decreased the pH of the pollen germination medium and, by consequence, of the cytoplasm [14]. This property is due to the high density of oxygen functional groups (e.g., carboxylic, epoxy, and oxydrilic) on the graphene lattice; the latter can also decrease the bioavailability of important cations for the development of pollen tube, e.g., calcium [15]. Regarding FLG, the authors hypothesized that the observed pollen germination decrease was due to an impairment of the germination pore and an imbalance of the intracellular reactive oxygen species (ROS).

The aforementioned in vitro experiments raised concerns about the possible negative effects of GRMs on one of the most important processes in nature: The sexual reproduction of seed plants. The success of fertilization of egg cell(s) strongly depends on pollination (i.e., the physical transfer of pollen from the male to the female part of a plant flower), stigma receptivity (i.e., the ability of the stigma to promote and support the germination of viable and compatible pollen [16]), and the interaction between the pollen grain and the stigmatic surface at the base of pollen recognition, germination, and pollen tube growth. The stigmatic surface receptivity can be affected by many environmental factors, such as temperature, rainfall, and parasites [17,18,19], as well as by exposure to xenobiotics [20,21,22]. Our hypothesis was that GRMs deposited on the stigmatic surfaces might interfere with the pollen–stigma interactions. This could occur due to chemical modifications of the substances present on the stigmatic surface or because the GRM flakes interpose themselves between pollen and the stigmatic papillae.

The physical interposition between pollen and stigma has been documented for airborne soil dust particles [23,24]. Hence, to properly understand if possible negative effects of GRMs on the stigmatic surface depend on their peculiar chemical-physical properties, a comparison with a naturally occurring material with similarly layered geometric structure seems to be appropriate. 

Phyllosilicates have a geometrical structure resembling that of GRMs, as they are made of silicon-based sheets a few atoms thick. One of these minerals, muscovite mica (MICA), has a layered structure consisting of two tetrahedral sheets on either side of an octahedral sheet composed of a honeycombed arrangement of hexagons of SiO_4_ tetrahedra [25]. Thus, its layered structure is rather similar to FLG, which is one of the most common and exploited GRMs [26]. MICAs account for up to 5.2% of the Earth’s crust [27], represent more than 6% of the nanometric fraction (<0.2 µm diameter) of loess soils, and are frequent components of terrigenous dust [28,29]. Furthermore, MICA is relatively inert from a (bio)chemical point of view, even at nanometric size [29,30]. For these reasons, using MICA as a comparison could help to discriminate if possible negative effects on the stigmatic surface occurring after exposure to GRMs are specifically related to the physical–chemical properties of these materials.

To verify the hypothesis of a possible impairment of plant reproduction caused by the interaction of GRMs with the pollen–stigma system, the effect of FLG and MICA on pollen viability, stigmatic surface integrity, pollen adhesion, and germination on the stigmatic surface were tested on *Cucurbita pepo* L. ssp. *pepo* ‘*greyzini*’ (the summer squash or zucchini), a model species for pollination studies [31] that is also economically important.

Understanding if FLG might have negative effects on plant reproduction processes and if these are caused by its peculiar properties would help us to take adequate measures to engineer safer-by-design GRMs.

## 2. Materials and Methods

### 2.1. NMs’ Preparation

FLG was prepared according to León et al. [29]: 7.5 mg of graphite (Bay Carbon Inc., Bay City, MI, USA) mixed with 22.5 mg of melamine (1,3,5-triazine-2,4,6-triamine, Sigma-Aldrich Chemie GmbH, Munich, Germany) were ball-milled at 100 rpm for 30 min using a planetary ball mill (Retsch PM 100, Retsch GmbH, Haan, Germany). The resulting solid mixture was suspended in 20 mL of bi-distilled water, sonicated for one minute, and dialyzed to remove melamine. The poorly exfoliated graphene was let to precipitate for five days and then removed from the suspension.

A sample of MICA (muscovite: KAl_2_(Si_3_Al)O_10_(OH)_2_ [32]) provided by the Department of Mathematics and Geoscience of the University of Trieste was pulverized using a tissue lyser (Retsch MM 400, Retsch GmbH, Haan, Germany). Two hundred mg of MICA lamellae were roughly broken with steel tweezers and then ball-milled at 30 Hz until glass-like lamellar structures were no longer visually recognizable.

### 2.2. NMs’ Characterization

FLG and MICA were subjected to physical–chemical characterization using qualitative and quantitative analytical techniques. FLG was analyzed with an inVia Raman Microscope (Renishaw, Wotton-under-Edge, UK) by drop-casting a FLG dispersion onto a Si wafer and dried on a hot plate. Overall, 30 Raman measurements were collected at different locations using a 532-nm laser bandwidth with an incident power of 1% (1 mW µm^−2^) and a 100× objective. 

Thermogravimetric analysis (TGA) of FLG was performed with a TGA Q50 (TA Instruments, New Castle, DE, USA) at 10 °C per minute under nitrogen atmosphere, from 100 °C to 800 °C.

Quantitative elemental analysis of FLG for C, H, N, and O was performed with a LECO CHNS-932 (LECO Corporation, St. Joseph, MI, USA) elemental analyzer, whereas a Bruker-S2PicoFox total reflection X-ray fluorescence (TXRF) spectrometer (Bruker Optics Inc., Billerica, MA, USA) was used for Al, Si, S, Cl, K, Ca, Ti, V, Fe, Ni, Cu, Zn, Br, and Sr.

Similarly, MICA was analyzed by X-ray powder diffraction (XRPD) with a Siemens D500 diffractometer (Siemens AG, Munich, Germany) using Cu Kα radiation (wavelength: 1.5406 Å), operated at 40 kV and 20 mA at room temperature in the range of 2*θ* from 5° to 30° (step 0.05° of 2*θ* and 1 s/step).

Qualitative elemental analysis was performed with Carl Zeiss Leo 1540 XB scanning electron microscope (SEM; Carl Zeiss, Oberkochen, Germany) equipped with an Everhart-Thornley secondary electron detector and in-column detector. Whole crystals (*n* = 4) or powdered (*n* = 6) MICA samples were attached to aluminum stubs and subjected to qualitative energy dispersive X-ray (EDX) analysis coupled to SEM. EDX spectra and maps were recorded using an Energy Dispersive Spectroscopy (EDS) System (Si[Li] detectors, liquid nitrogen cooled, total area 10 mm^2^; EDAX, Mahwah, NJ, USA). The EDX spectra were collected with 60 s acquisition time; for the maps, 10- to 30-min acquisitions were used. Electron beam energy of 3 KeV was used for morphological characterization, while 6 and 12 KeV were used for EDX analysis. 

Furthermore, both NMs were characterized morphologically to determine their lateral dimension and thickness. High-resolution transmission electron microscope (HRTEM) observations were taken with a JEM 2100 (JEOL Ltd., Tokyo, Japan). Stable dispersions of both materials were drop-casted on nickel grids (3.00 mm, 200 mesh), dried under vacuum, and observed at an accelerating voltage of 100 kV. Lateral dimension distribution of NMs was calculated with ImageJ1 software (ImageJ, version 1.52a; image processing program, The National Institutes of Health, Bethesda, MD, USA, 1997). NMs’ thickness was measured with an atomic force microscope (AFM) under ambient conditions using Multimode V7.30 (Veeco Instruments Inc., Plainview, NY, USA) with a NanoScope V controller (Digital Instruments, Tonawanda, NY, USA) working on tapping mode with a silicon tip (HQ:NSC15/Al BS, MikroMasch OÜ, Tallin, Estonia) at a working frequency of 235 kHz and a nominal force constant of 40 Nm^−1^. Height and phase images were simultaneously obtained. The samples were prepared with an aliquot of 10 µL of a 100 µg/mL NMs’ dispersion (previously sonicated for 10 min) by spin coating on silicon surfaces.

### 2.3. Cultivation and Growth of Cucurbita Pepo L.

*Cucurbita pepo* L. ssp. *pepo* ‘*greyzini*’ is a monoecious, entomophilous therophyte with short internodes and with indeterminate growth and reproduction [33,34]. Male flowers start to appear approximately one month before female ones, c. 60–75 days after seed germination, and are produced for the whole remaining growth season. Each flower develops individually and lasts for a few hours after anthesis, which generally occurs around 6:00 a.m. Plants of *C. pepo* were greenhouse cultivated in the Botanical garden of the University of Trieste from March to May 2019 and then transplanted in open-field conditions. Plants were watered once a day with a ground-based automatic irrigation system, fertilized once a week, and treated with antimycotics once a week (Azupec 80WG, Ascenza Agro, Torres Vedra, Portugal or Jupiter WG, Isagro Spa, Adria, Italy). For the experiments, male and female flowers were collected the day before anthesis and kept hydrated overnight in the laboratory (21 °C) to prevent pollination.

### 2.4. Effects of NMs on C. Pepo Reproduction

In the present work, effects of manufactured FLG and, for comparison, natural MICA NMs were evaluated on different steps of the *C. pepo* reproduction process, testing either male or female structures and their interactions. Pollen viability, stigmatic surface integrity, pollen adhesion, and germination onto stigmatic surfaces were used as proxies.

### 2.5. Pollen Viability

Pollen of *C. pepo* is of the recalcitrant type, i.e., it is partially hydrated and metabolically active at stamen dehiscence. This pollen dehydrates in a few hours, progressively losing viability [35,36]. Pollen viability, eventually in the presence of FLG and MICA, was assessed by the fluorescein diacetate (FDA) (Sigma-Aldrich Chemie GmbH, Munich, Germany) fluorochromatic reaction (FCR) [37].

FDA was dissolved in Brewbaker and Kwack’s (BK) culture medium (15% *w/v* of sucrose) [38], to a concentration of 2 mg mL^−1^, while 100 µL of FDA solution were poured over a microscope glass slide and then spiked with a small amount (the tip of a needle) of pollen. After 10 min of dark incubation at room temperature, FDA fluorescence was visualized using a Zeiss Axioplan microscope (Carl Zeiss, Oberkochen, Germany) equipped with a Kiralux CS505CU camera (Thorlabs Inc., Newton, NJ, USA). To test the effect of FLG and MICA on pollen viability, viable pollen was initially pooled and then divided into three aliquots. One was kept pristine (CTRL) whereas the other two were mixed with FLG and MICA (treated), respectively, in a ratio of 0.5 and 2 mg NM per g^−1^ of pollen (fresh weight). Evaluation of viability percentage was performed—counting at least 200 pollen grains for each sample on pollen collected from at least seven flowers, each from a different plant—if the initial viability was higher than 70%. Measurements were carried out after 15, 45, 90, 180, and 360 min of exposure.

### 2.6. Stigma Exposure to NMs

The female flowers of *C. pepo* bear a single pistil with three stigmas, each made of two lobes [33].

To reduce variability, exposure to NMs was carried out, with 1 mg of FLG and MICA dry powder administered to the stigmatic surface of two of the three stigmas, respectively (treated stigmas), of each flower (*n* = 3–6). Treated stigmas were gently and homogeneously coated using a brush until the stigmatic surface was completely covered by the NMs (Figure S1); the surface of the third stigma was instead brushed only with a clean paintbrush, as control (CTRL). Stigmas were then kept at laboratory conditions (dim light and 21 °C) for three hours before SEM analysis. After this period, a further set of CTRL and treated stigmas were hand-pollinated with fresh pollen (viability >85%) using a paintbrush in order to test the effects of the NMs on pollen detachment and in vivo germination. For the detachment experiments, 5 mg of pollen (estimated 4105 ± 931 pollen grains) were brushed all over the stigmatic surface, whereas for the germination experiments, 3 ± 0.5 mg of pollen (estimated 2407 ± 541 pollen grains) were applied over a stigma spot of 16 mm^2^ using a squared (4 × 4 mm), greaseproof, paper frame. Afterwards, pollen was let to germinate at laboratory conditions (see above) for 40 min. Pollen adhesion to the stigmatic surface and germination rate were then assessed (see infra). Both experiments were replicated three to six times.

### 2.7. NMs’ Effect on the Stigmatic Surface

Three CTRL and treated stigmas were excised, attached to aluminum stubs, and observed using a SEM (Quanta250 SEM, FEI, Oregon, USA) operated in environmental mode (ESEM) collecting secondary electrons. The working distance was adjusted to obtain the suitable magnification, the accelerating voltage was 30 kV, and the pressure was set at 90 Pa. The entire stigmatic surface of each sample was examined to find potential signs of damage by taking 20 images per sample.

### 2.8. Pollen Detachment from the Stigmatic Surface

The presence of a very thin, powdery material might interfere with the adhesion of pollen deposited over the stigmatic surface, increasing pollen detachment from the latter and then decreasing the pollen germination rate. To evaluate both effects, we used the aniline blue staining protocol of Herburger and Holzinger [39]. After the germination period, CTRL and treated stigmatic lobes were immersed for 20 min in 5 mL of 8 N NaOH solution at 60 °C. Afterwards, NaOH was removed and the lobes were washed three times with 5 mL of dH_2_O for five minutes to remove remaining NaOH. The NaOH solution and dH_2_O used for washing were pooled separately for each lobe and centrifuged at 6000× *g* to pellet-detached pollen. Following this, 18 mL of the supernatant were discarded and a 200-µL aliquot of the remaining suspension was observed at the light microscope to count the pollen and, thus, estimate those detached from the stigmatic lobes.

### 2.9. In Vivo Pollen Germination on Stigma

After the abovementioned washing procedure, the stigmas were embedded with a mounting medium (Killik, Bio-Optica, Milano, Italy) and then sliced with a Leica CM 1510 S cryostat (Leica microsystems^®^, Wetzlar, Germany) to obtain 150-µm-thick cross-sections. These (*n* = 28 ± 3) were transferred over polysine glass slides (Menzel Gläser™, Thermo Fisher Scientific, Waltham, MA, USA), dehydrated at room temperature, and then washed with dH_2_O to remove the mounting medium. The slices were then stained with aniline blue for five minutes, washed to remove the excess of stain, and observed with the abovementioned epi-fluorescence microscopy system. Pollen germination over the stigmatic surface was evaluated on the digital images were acquired with the Fiji software. For each sample, 193 ± 32 pollen grains were counted from randomly chosen images to calculate the percentage of germinated pollen (approx. 1100 pollen grains counted per treatment).

### 2.10. Statistical Analysis

Data calculations were performed using Microsoft^®^ Excel^®^ 2016 (Excel^®^, version 16.0.12527.206120, Microsoft 365^®^; version 18.2006.1031.0; Microsoft, Redmond, Washington, DC, USA, 2016). The add-on package PERMANOVA+ [40] of Primer software (Primer, version 7, software for univariate and multivariate statistical analysis, Primer-E Ltd, Plymouth Marine Laboratory, Plymouth, UK, 2015) was used to perform multi-dimensional scaling (MDS), permutational multivariate analysis of variance (PERMANOVA) routine, and Monte Carlo test [41].

## 3. Results and Discussion

### 3.1. NMs’ Characterization

The physical-chemical analyses of FLG confirmed the good quality of the material: The Raman spectrum showed the classical two most intense peaks of FLG appearing at ~1580 (G band) and ~2700 (2D band) cm^−1^ (Figure S2a). The average ratio between the intensity (I) of 2D and G bands (I(2D)/I(G)) was 0.43, consistent with the value (<1) usually assigned to FLG [42,43]. The occurrence of a further band with peak at ~1345 cm^−1^ (D band) (Figure S2a) suggests the existence of some defects on the graphene lattice, likely related to gaps at the margin of nanometric sheets [44]. Nevertheless, the average I(D)/I(G) was 0.46, indicating a low level of defects. This was corroborated by both TGA [45] and the elemental composition (Figure S2c, Table S1); indeed, FLG was made of C for more than 95%.

FLG flakes had lateral dimensions ranging from 50 to 950 nm, with an average of 509 ± 233 nm (Figure S2e); flakes with a lateral dimension smaller than 200 nm were 7.4% of the total (Figure S2g). AFM measurements revealed that FLG NM had a thickness from 3 to 12 nm with an average of 5 ± 2 nm (Figure S3). However, data from literature revealed large disparity between AFM measurements for a graphene layer, with thicknesses ranging from 0.35 nm to 1 nm relative to the SiO_2_ substrate [46,47,48]. This is mainly due to the difficulty in depositing FLG from solvents without observing reaggregation and to experimental artefacts and contaminations that may affect AFM measurements. For this reason, Raman spectroscopy measurements of FLG thickness are preferable as it is commonly accepted that the thickness of few-layer graphene nanosheets is reflected in the shapes of their 2D Raman bands (around 2700 cm^−1^) [49]. Accordingly, applying the equations of Coleman and collaborators [50], Raman results corroborated by AFM measurements suggested that our few-layer graphene consisted of flakes with an average thickness of 3–4 layers [49].

MICA was selected for its similarity to FLG, having a planar structure a few atoms thick and an analogous outstanding surface/volume ratio [51] but differing in elemental composition [32]. XRPD analysis confirmed the identity of the mineral sample, showing peaks at 8.8°, 17.8°, and 26.8° (Figure S2b) [52]. The EDX spectra revealed the presence of Si, Al, O, and K of original muscovite with substituents usually present in its skeleton (Na, Mg, Ca, Fe) and further elements less common in phyllosilicates (C, F, and Ne) (Figure S2d) [53] but with scarce to no toxicity on plant tissues [54]. The presence of C might be related to a residual of the stub used in sample analysis (see Section 2.2), whereas that of Ne appeared only in one case. F and Ne signals disappeared after the milling process, whereas Fe traces occurred in all the spectra (Figure S2d), seemingly as a consequence of the steel balls’ abrasion during the milling process. The MICA powder was a mixture of micro- and nanocrystals. The latter accounted for c. 80% of the total and had a maximum lateral size dimension of 344 ± 249 nm (Figure S2f). Of these, 32.5% had a lateral dimension lower than 200 nm (Figure S2h). MICA nanocrystals had a thickness ranging from 3 to 50 nm with an average of 16 ± 13 nm (Figure S3). For this material, the height of a monolayer was around 1 nm [55], therefore the AFM analysis evidenced that MICA nanocrystals were thicker than FLG.

### 3.2. Effects of Planar Materials on Pollen Viability

**Figure 1:**
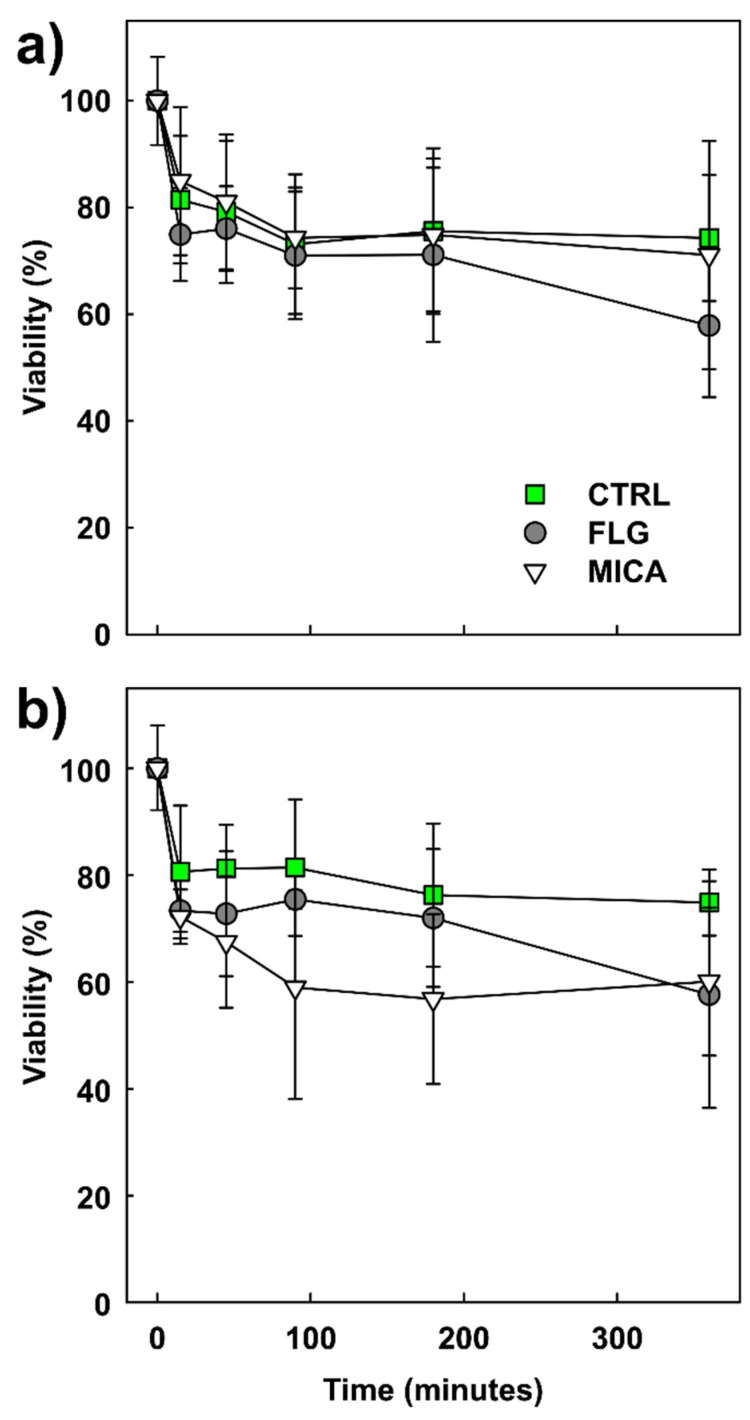
(**a**) Viability of *Cucurbita pepo* L. pollen in untreated (CTRL) and few-layer graphene (FLG)- or muscovite mica (MICA)-treated samples during six hours’ incubation at 2 mg of nanomaterials (NMs) per g of pollen; (**b**) the same at 0.5 mg of NMs per g of pollen. Pollen viability is expressed as a percentage of the viability of freshly harvested pollen (*n* = 200). Values are means ± s.d. (*n* = 3–4). Statistical analysis did not reveal significant differences among treatments for any time points (PERMANOVA, P-perm > 0.05, for both concentrations; for more details, see Table S2).

The pollen is the plant microgametophyte and has the fundamental role of carrying the male genetic material intact to the ovule where the fertilization of the egg cell(s) takes place. Therefore, any harmful effect on pollen might result in reduced success of reproduction [56,57]. Viability of CTRL and treated pollen progressively decreased over time (Figure 1). No statistically significant differences were observed among treatments and time points (Table S2). This phenomenon is in accordance with previously reported data for this species, which has recalcitrant pollen [34,36]. Neither FLG nor MICA affected *C. pepo* pollen viability. In the case of FLG, similar results were reported for the pollen of *C. avellana* and *N. tabacum* [14,15]. There are few studies reporting damage to plant cells or tissues by FLG exposure, ascribing these effects to the hardness of the FLG flakes that can cut through membranes [58] and, seldom, cell walls [59]. In the case of MICA, the only (eco)toxicological studies available focus on animals and show minor damage both for in vitro and in vivo conditions [29,30], as well as for MICA nanocrystals [29]. However, pollen possesses a thick barrier, i.e., the double pollen wall, consisting of a thin, delicate, inner wall of unaltered cellulose (endospore or intine) and a tough, resistant, two-layered outer wall (the exospore or exine) composed largely of an exceptionally resistant biopolymer, sporopollenin, eventually covered with waxes, glycolipids, and proteins [60]. As previously shown [15], the highly specialized pollen wall can efficaciously prevent internalization of FLG or other materials as MICA, even when at a nanometric size.

### 3.3. Interaction of MICA and FLG with the Stigmatic Surface

The stigma strictly controls the conditions for pollen self-recognition (proteins, lipids, etc.) and germination, stimulating or inhibiting it through the control of osmolarity, pH, Ca^2+^ concentration, etc. [17,61,62]. Consequently, any defect or impairment of the stigmatic surface can affect the pollination process and, eventually, the plant reproduction. Indeed, a damaged stigmatic surface could release intracellular fluids containing stabilizing and/or reducing substances, as observed in the leaves’ extracts of *C. pepo* [63]. These substances may change not only the stigmatic environment but also the NMs’ properties, either increasing or reducing possible toxic mechanisms.

**Figure 2:**
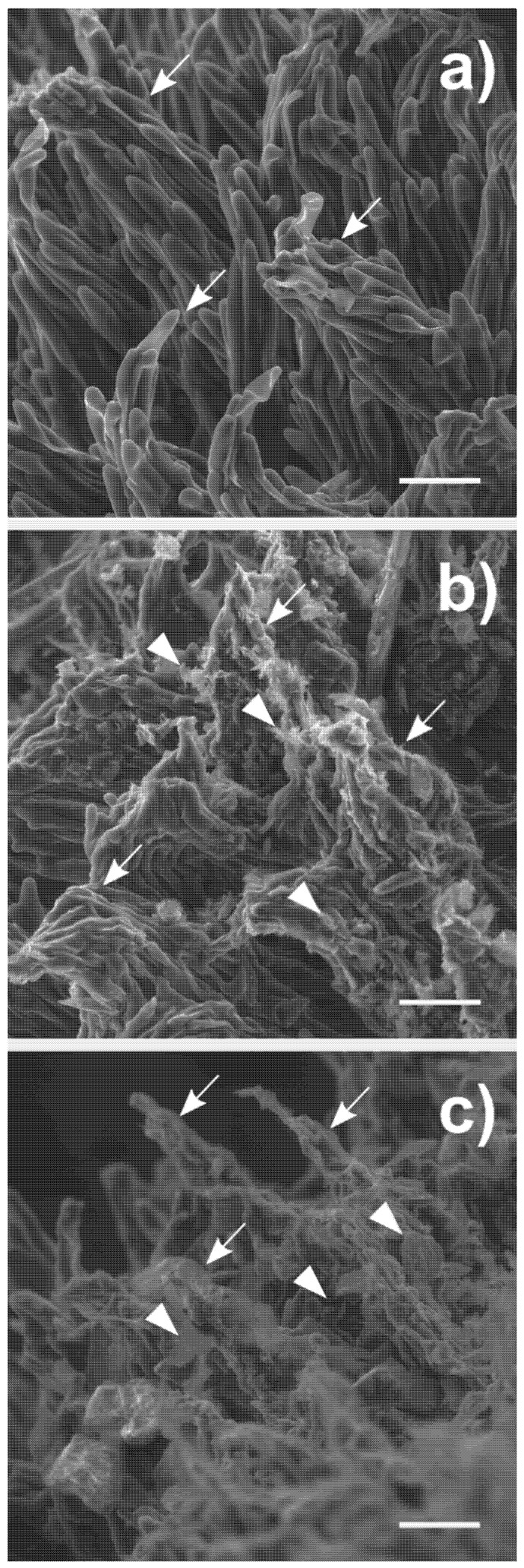
(**a**) SEM micrographs of stigmatic surfaces of *Cucurbita pepo* L. female flowers treated for three hours without nanomaterials (CTRL); (**b**) with 1 mg of few-layer graphene (FLG); (**c**) with 1 mg of muscovite mica (MICA). Stigmatic papillae are indicated with arrows, nanomaterials with arrowheads. Bars = 100 µm. For further images, see Figure S4.

The stigmatic surface of *C. pepo* is a complex structure made of finger-like-shaped cells forming clump-like structures, the so-called stigmatic papillae (Figure 2a and Figure S4a,b). ESEM observations revealed that NMs deposited on the stigmas did not affect the integrity of their surface, as the papillae maintained the original shape or were found just slightly agglutinated (Figure 2b,c and Figure S4c,d); no wilted cells or cytoplasmic leachates were observed. Studies on the effect of depositions over the stigmatic surface are very few, and only Zhang and collaborators [23] tested the deposition of insoluble soil particulate matter. These authors reported that the exposure of *Pistacia vera* (pistachio plant) flowers to soil dust caused wilting of the stigma and collapse of the stigmatic papillae. A direct comparison between these observations and the results reported here is difficult because the only characterization of the dust applied to the *P. vera* flowers was the size of the dust particles (20 µm in diameter), but not their physicochemical properties. It is widely recognized that salts or toxic elements adsorbed on particulate surfaces as well as the presence of functional groups, dimensions, and geometry of particles are very important factors in determining toxic effects [64]. One of the main reasons for the different results obtained may be related to the biology of the species tested. *C. pepo* has a dry stigma [65], i.e., it does not secrete any fluid on its surface when ripened, whereas pistachio has a wet stigma [65]. The presence of a liquid layer coating stigmatic papillae might dissolve salts, causing dissociation of weakly bound ions from the particle surfaces, making these more reactive toward the surrounding environment. Indeed, when the stigmas were coated with dust contaminated with an herbicide in the abovementioned work, the papillae were completely decomposed [23].

### 3.4. Pollen–Stigma Adhesion in the Presence of FLG and MICA

**Figure 3:**
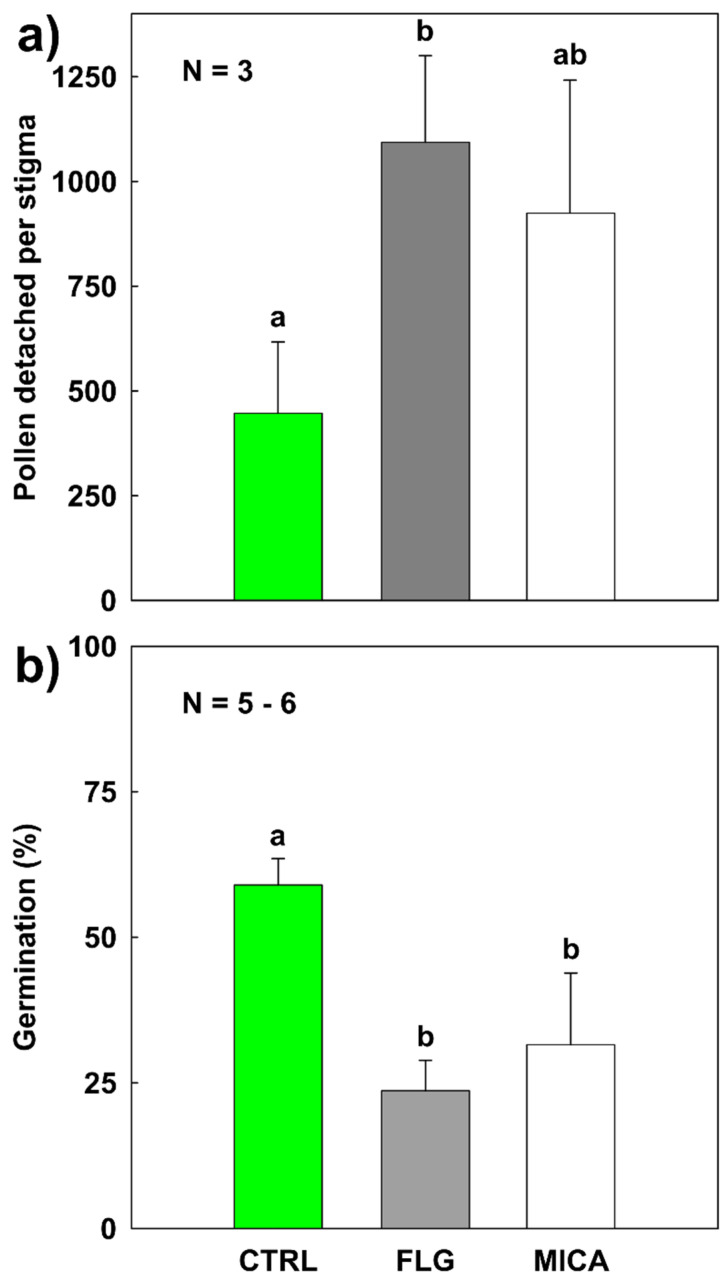
(**a**) Pollen detachment from pristine (CTRL) stigmatic surface of *Cucurbita pepo* L. or pretreated with 1 mg of few-layer graphene (FLG) and muscovite mica (MICA) for three hours. Pollen detachment was evaluated after 40 min from pollination on the washing solutions derived from the application of the aniline blue staining protocol (for more details, see chapter 2.8); (**b**) percentage of germinated pollen still adhered on stigmatic lobes treated as in (a), values are means ± s.d. Statistically different groups are marked with different letters (PERMANOVA, Monte Carlo post hoc test; for more details, see Table S3).

The last step of pollination is the adhesion of the pollen grain to the stigmatic surface [17,62]. Powdery materials deposited on stigmas might interfere with pollen adhesion, although the *C. pepo* pollen is very rich in pollenkitt, a viscous substance that glues the grains to the papillae [56,66]. In each pollen load, around 4000 pollen grains were brushed on the stigmatic surface of each sample. In CTRL samples, not all the loaded pollen adhered tightly to the stigmatic surface, as 447 ± 170 (c. 10%) pollen grains detached (Figure 3a) during the washing steps (see Section 2.7). The number of grains detached from FLG- and MICA-treated samples were 1093 ± 207 and 924 ± 317, respectively, statistically increasing in respect to CTRL only in the case of FLG (CTRL vs. FLG pairwise: *p* = 0.016, Monte Carlo post hoc test for pairwise comparisons) (Table S3). However, no statistically significant differences (pairwise *p* > 0.4) were found between MICA- and FLG-treated samples. In other words, the presence of a powdery material decreased the pollen adhesion to the stigmatic surface independently of the chemical composition/properties of the materials.

Though this pollination step is very important [17,62], little is known about pollen–stigma bonding and the factors affecting it [67]. Indeed, the few available studies focused only on the strength of this bond. Furthermore, most works that studied the effect of dust-like deposition on plant reproduction tended to focus on fruit production [24,68,69] rather than on the stigma–pollen interaction [22,23]. *C. pepo* is a model plant for pollination experiments [31]; nonetheless, its flowers have a short opening time [34]. Thus, it is improbable that significant amounts of airborne NMs might land onto its stigmatic surface. The situation may change for long-lasting flowers that can endure up to one month before wilting, such as anemophilous species with stigma unprotected by sepals and specialized in intercepting aerodisperse pollen [70]. These flowers might be more sensitive to airborne NMs. Indeed, the longer a flower remains exposed to air, the higher the chance that stigmas intercept NMs to amounts that could potentially decrease the chances of fertilization success [71].

### 3.5. In Vivo Pollen Germination on Stigma Exposed to NMs

**Figure 4:**
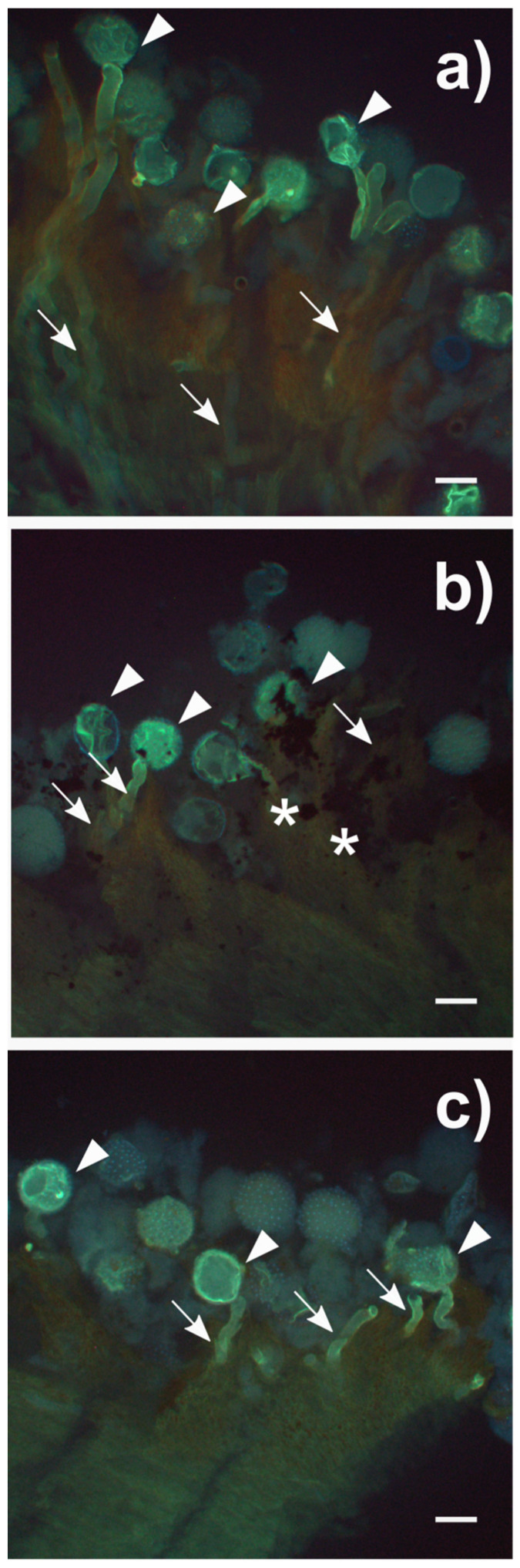
(**a**) Fluorescence micrographs of stigmatic surfaces of hand-pollinated *Cucurbita pepo* L. female flowers treated for three hours without nanomaterials (CTRL); (**b**) with 1 mg of few-layer graphene (FLG); (**c**) with 1 mg of muscovite mica (MICA). Aniline blue staining protocol was applied 40 min after pollination (for more details, see text). Pollen tubes are indicated with arrows, pollen grain with arrowheads, and FLG with asterisks. Bars = 100 µm.

The presence of powdery materials on the stigmatic surface may also interfere with further steps of the pollen–stigma interaction, e.g., by modifying chemical signals inducing pollen germination. The germination rate on the stigma of CTRL samples was 59 ± 5% (Figure 3b), very similar to that reported for this species (71 ± 17%) [70]. However, germination on FLG- and MICA-treated samples significantly decreased to 24 ± 5% and 32 ± 12%, respectively (pairwise *p* > 0.002, Monte Carlo post hoc test for pairwise comparisons). Similarly to the pollen detachment results, FLG had a slightly stronger effect on germination than MICA, but the difference was not statistically significant (PERMANOVA *p* > 0.3, Table S3). After three hours, the pollen tubes generally penetrated deep into the pristine stigmatic tissues (Figure 4a), far less in those treated with FLG and MICA (Figure 4b,c).

Our results are in accordance with the findings of Candotto Carniel et al. [15]: Both studies indicate that FLG caused a decrease in pollen germination. However, material administration modalities (liquid suspension vs. dry application, in vitro vs. in vivo, FLG concentrations) and pollination biology of the tested species (*C. pepo* vs. *C. avellana*) were quite different. Furthermore, the similar decrease of pollen germination observed on MICA-treated samples suggests that the underlying mechanism is also different and not strictly related to the chemical properties of the material. Indeed, our NMs affected neither pollen viability nor stigmatic surface integrity. In addition, possible chemical interactions with NMs, such as release or binding of ions as reported for Ag-, Pd-, and Fe-oxide nanoparticles [15,72,73,74,75], would be limited as *C. pepo* stigma is of the dry type. These arguments, together with the results on pollen detachment, allow us to hypothesize that NMs physically hinder the contact between pollen and stigma, affecting the signaling mechanisms and, consequently, the germination stimulus. In other plants, the removal of the substances at the base of this stimulus resulted in partial or total inhibition of in vivo germination [17,76,77].

## 4. Conclusions

In this study, the possible impact of FLG was verified on a biological process essential for terrestrial ecosystems, i.e., the sexual reproduction of seed plants. The possible negative effects of this material were verified on the pollen viability, stigma integrity, and pollen adhesion and germination on the stigmatic surface of a model species in pollination studies, *C. pepo*. Importantly, we used MICA for comparison, a phyllosilicate with a layered structure similar to GRMs that was conveniently treated to be dimensionally similar to FLG. Neither pollen viability nor stigmatic surface integrity were affected by the tested NMs. Nonetheless, both of them equally reduced pollen adhesion and germination on the stigma. These effects were likely caused by the interposition of flakes and/or crystals between pollen and stigma, affecting, de facto, their interaction. Our samples were exposed to relatively high amounts of NM powders unlikely to occur in the environment. However, the use of such amounts allowed possible effects of NM depositions on stigmas to be highlighted, paving the way for future investigations on anemophilous species. Importantly, in this study, the comparison with MICA highlighted that FLG can be considered as safe as a naturally occurring planar NM, not uncommon in soil particulate matter. However, before excluding FLG from the ecotoxicologically relevant substances for seed plants, biodistribution and transformation in the plant body [78,79] still need to be thoroughly investigated.

## Supplementary Materials

The following are available online at https://www.mdpi.com/2079-4991/10/9/1877/s1, Figure S1: Nanomaterials’ characterization, Figure S2: AFM characterization, Table S1: Elemental characterization of FLG, Figure S3: Stigmatic surfaces treated with FLG, MICA, and control, Table S2: PERMANOVA results of pollen viability, Table S3: PERMANOVA results of pollen detachment from the stigma and % of pollen in in vivo germination, Figure S4: SEM micrograph of stigmatic papillae.
