# Supplementary material for: Effects of Few-Layer Graphene on the Sexual Reproduction of Seed Plants: An In Vivo Study with Cucurbita pepo L
Source: Nanomaterials (Basel). 2020 Sep 19;10(9):1877. doi: 10.3390/nano10091877 (PMC7560101; doi:10.3390/nano10091877)
Supplement: Supplementary file 1 [file nanomaterials-10-01877-s001.pdf]

nanomaterials

Supplementary Materials

Effects of Few-Layer Graphene on the Sexual Reproduction of Seed Plants: An In Vivo Study with *Cucurbita pepo* L.

Davide Zanelli [1], Fabio Candotto Carniel [1,*], Marina Garrido [2], Lorenzo Fortuna [2], Massimo Nepi [3], Giampiero Cai [3], Cecilia Del Casino [3], Ester Vázquez [4,5], Maurizio Prato [2,6,7] and Mauro Tretiach [1]

1. Department of Life Sciences, University of Trieste, via L. Giorgieri 10, I-34127 Trieste, Italy; davide.zanelli@phd.units.it (D.Z.); fcandotto@units.it (F.C.C.); tretiach@units.it (M.T.)
2. Department of Chemical and Pharmaceutical Science, University of Trieste, via L. Giorgieri 1, I-34127 Trieste, Italy; mgarrido@units.it (M.G.); lfortuna@units.it (L.F.); prato@units.it (M.P.)
3. Department of Life Sciences, University of Siena, via P. A. Mattioli 4, I-53100 Siena, Italy; massimo.nepi@unisi.it (M.N.); giampiero.cai@unisi.it (G.C.); cecilia.delcasino@unisi.it (C.D.C.)
4. Department of Organic Chemistry, Faculty of Chemical Science and Technology, University of Castilla-La Mancha, Av. Camilo José Cela, s/n, E-13005 Ciudad Real, Spain; ester.vazquez@uclm.es
5. Instituto Regional de Investigación Científica Aplicada (IRICA), Universidad de Castilla-La Mancha, E-13071 Ciudad Real, Spain
6. Center for Cooperative Research in Biomaterials (CIC biomaGUNE), Basque Research and Technology Alliance (BRTA), Paseo de Miramón 182, 20014 Donostia San Sebastián, Spain
7. Basque Foundation for Science, Ikerbasque, 48013 Bilbao, Spain

* Correspondence: fcandotto@units.it; Tel.: +39-(04)-05583879

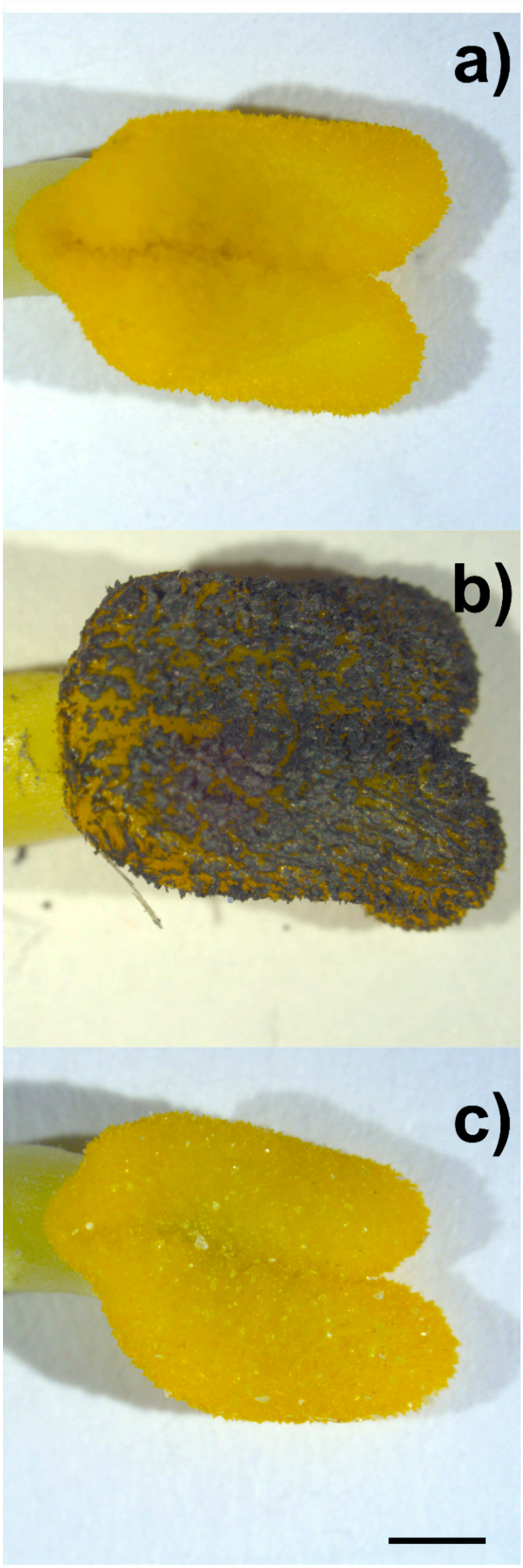

Figure S1. (**a**) *Cucurbita pepo* L. stigmas treated without nanomaterials (CTRL); (**b**) with 1 mg of few-layer graphene (FLG); (**c**) with 1 mg of muscovite mica (MICA). Bar = 2 mm.

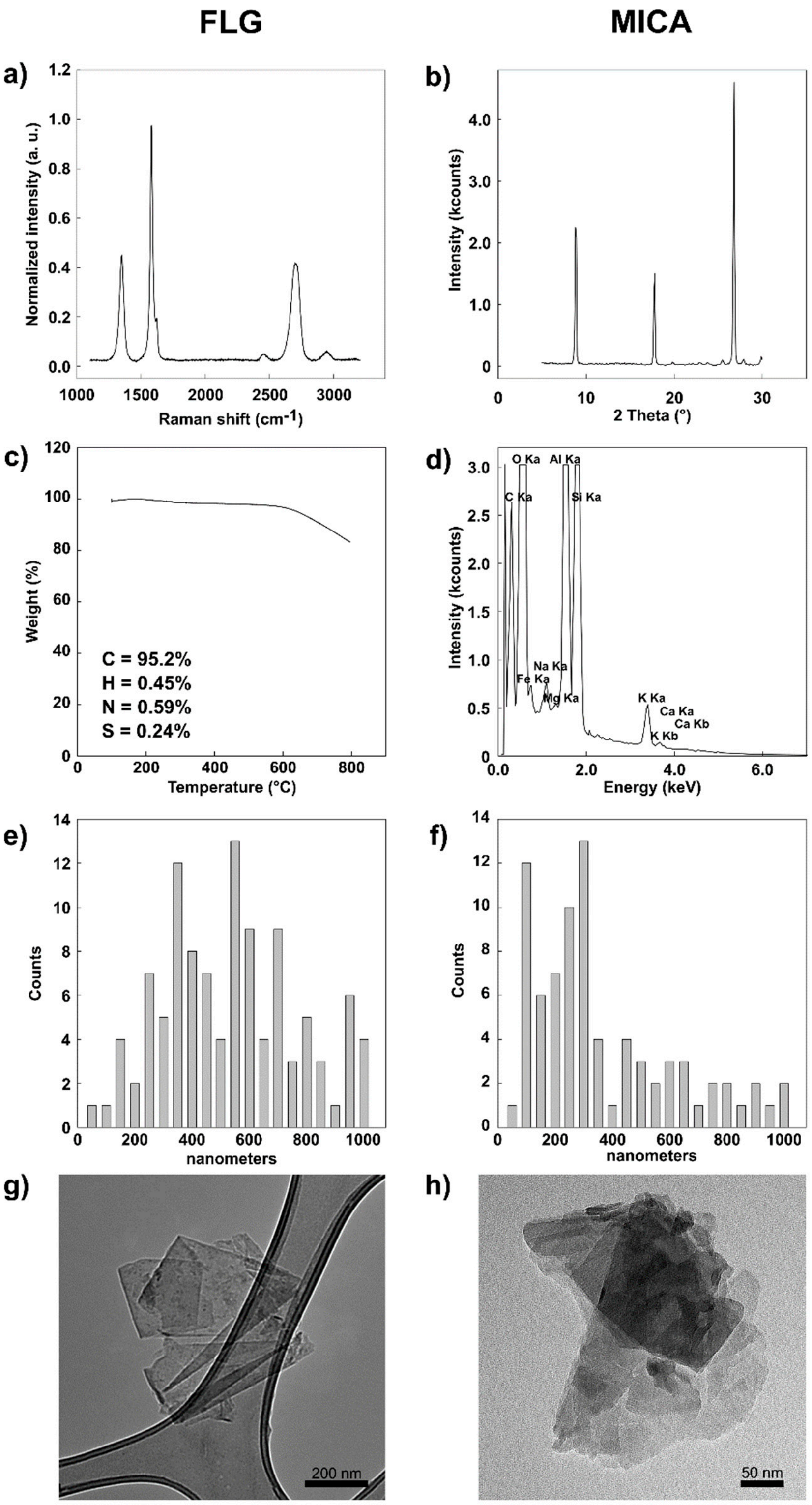

Figure S2. Physical–chemical characterization of few-layer graphene (FLG, left column) and muscovite (MICA, right column): (**a**) average Raman spectra; (**b**) X-ray powder diffraction; (**c**) thermogravimetric and elemental analysis; (**d**) energy dispersive X-ray (EDX) analysis; (**e**, **f**) lateral size distribution of sheets (n > 80); (**g**) representative TEM image of FLG; (**h**) representative TEM image of MICA.

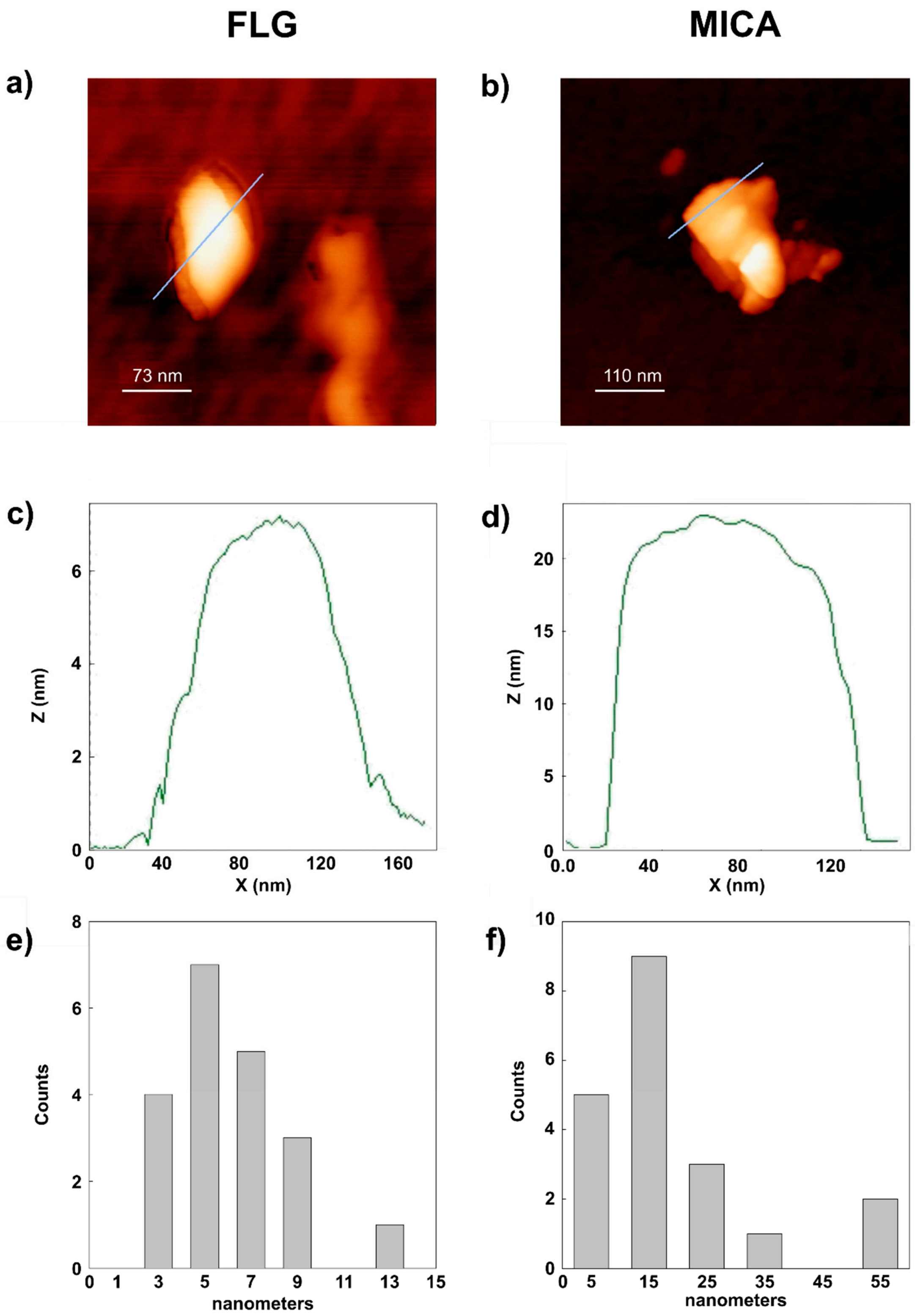

Figure S3. Atomic force microscopy (AFM) characterization of few-layer graphene (FLG, left column) and muscovite (MICA, right column): (**a**) representative AFM images of FLG flakes; (**b**) representative AFM images of MICA nanocrystals; (**c**) height profile of (a); (**d**) height profile of (b); (**e**) thickness distribution of FLG flakes; (**f**) thickness distribution of MICA nanocrystals (n = 20 for (e) and (f)).

Table S1. Total reflection X-ray fluorescence (TXRF) elemental analysis of few-layer graphene (FLG).

Element	Line	Concentration mg/L	Sigma/ mg/L	RSD/ %	LLD/ mg/L	Net Area	Background	Chi
**Al**	K12	0.44	0.18	42.0	0.38	121	1224	1.44
**Si**	K12	69.98	0.41	0.6	0.16	44845	1214	4.24
**S**	K12	0.905	0.029	3.3	0.044	2130	1189	1.81
**Cl**	K12	0.039	0.013	34.2	0.027	149	1225	0.88
**K**	K12	0.089	0.007	7.3	0.012	755	1105	0.97
**Ca**	K12	0.503	0.009	1.8	0.010	5154	1184	0.54
**Ti**	K12	6.396	0.025	0.4	0.005	125219	1245	1.50
**V (IS)**	K12	2.000	0.011	0.6	0.003	49353	795	1.66
**Fe**	K12	0.019	0.001	5.0	0.001	894	523	0.86
**Ni**	K12	0.014	0.001	4.7	0.001	966	497	0.99
**Cu**	K12	0.017	0.001	3.8	0.001	1361	586	0.84
**Zn**	K12	0.054	0.001	1.6	0.001	5071	465	1.38
**Br**	K12	0.002	0.000	10.6	0.000	353	507	1.26
**Sr**	K12	Not det.			0.001	1	1353	3.78

Table S2. Permutational multivariate analysis of variance (PERMANOVA) comparison of *Cucurbita pepo* L. pollen viability of untreated (CTRL) and few-layer graphene (FLG)- or muscovite mica (MICA)-treated samples at 2 and 0.5 mg per g (fresh weight) of pollen after 15 (T1), 45 (T2), 90 (T3), 180 (T4), and 360 (T5) minutes. Values are reported as mean ± s.d. N: Number of replicates per single treatment; Pseudo-F: Statistic computed for the single factor (for more details see text) by PERMANOVA; P(perm): Permutation *p*-value; statistically different groups (Monte Carlo post hoc test) at the same time point are marked with different letters [for P(perm) < 0.05].

**Treatment**		Viability (%)												**Pseudo-F**	**P(perm)**
	**N**	**T0**	**T1**		**T2**		**T3**		**T4**		**T5**				
**at 2 mg g^{-1}**														2.3219	0.1021
	4	100 ± 8.1													
CTRL	4		80.6 ± 12.4	*a*	81.3 ± 8.2	*a*	81.4 ± 12.8	*a*	76.3 ± 13.4	*a*	74.9 ± 6.2	*a*			
FLG	4		72.2 ± 5.1	*a*	67.5 ± 12.3	*a*	59.0 ± 20.9	*a*	56.8 ± 15.9	*a*	60.1 ± 13.9	*a*			
MICA	4		73.4 ± 4.0	*a*	72.8 ± 11.7	*a*	75.5 ± 1.8	*a*	72.0 ± 12.9	*a*	57.6 ± 21.2	*a*			
**at 0.5 mg g^{-1}**														0.54628	0.6065
	4	100 ± 8.5													
CTRL	4		81.5 ± 12.0	*a*	79.2 ± 13.4	*a*	73.1 ± 13.1	*a*	15.6 ± 15.5	*a*	74.3 ± 11.8	*a*			
FLG	4		74.9 ± 8.7	*a*	76.0 ± 7.9	*a*	71.0 ± 12.0	*a*	71.1 ± 16.3	*a*	57.9 ± 13.4	*a*			
MICA	3		84.9 ± 13.9	*a*	81.0 ± 12.7	*a*	74.3 ± 9.4	*a*	74.9 ± 14.3	*a*	71.1 ± 21.4	*a*			

Table S3. Permutational multivariate analysis of variance (PERMANOVA) comparison of pollens detached (Detachment) and pollen germination percentage (Germination) on stigmas from pristine (CTRL) stigmatic surface of *Cucurbita pepo* or pretreated with 1 mg of few-layer graphene (FLG) and muscovite (MICA) for 3 h. Pollen detachment was evaluated after 40 min from pollination on the washing solutions derived from the application of the aniline blue staining protocol; after the same period, pollen germination was assessed on cross-sections of washed stigmas (for more details, see text). Values are reported as mean ± s.d.; N: Number of replicates per single treatment; Pseudo-F: Statistic computed for the single factor by PERMANOVA; P(perm): Permutation *p*-value; statistically different groups (Monte Carlo post hoc test) are marked with different letters [for P(perm) < 0.05].

**Treatment**	**Pseudo-F**	**P(perm)**		**Detachment**		**Pseudo-F**	**P(perm)**		**Germination**	
	5.883	0.059	**N**			28.21	0.001	**N**		
CTRL			3	447 ± 170	*a*			6	59.0 ± 4.51	*a*
FLG			3	1093 ± 207	*b*			5	23.7 ± 5.24	*b*
MICA			3	924 ± 317	*ab*			6	31.6 ± 12.3	*b*

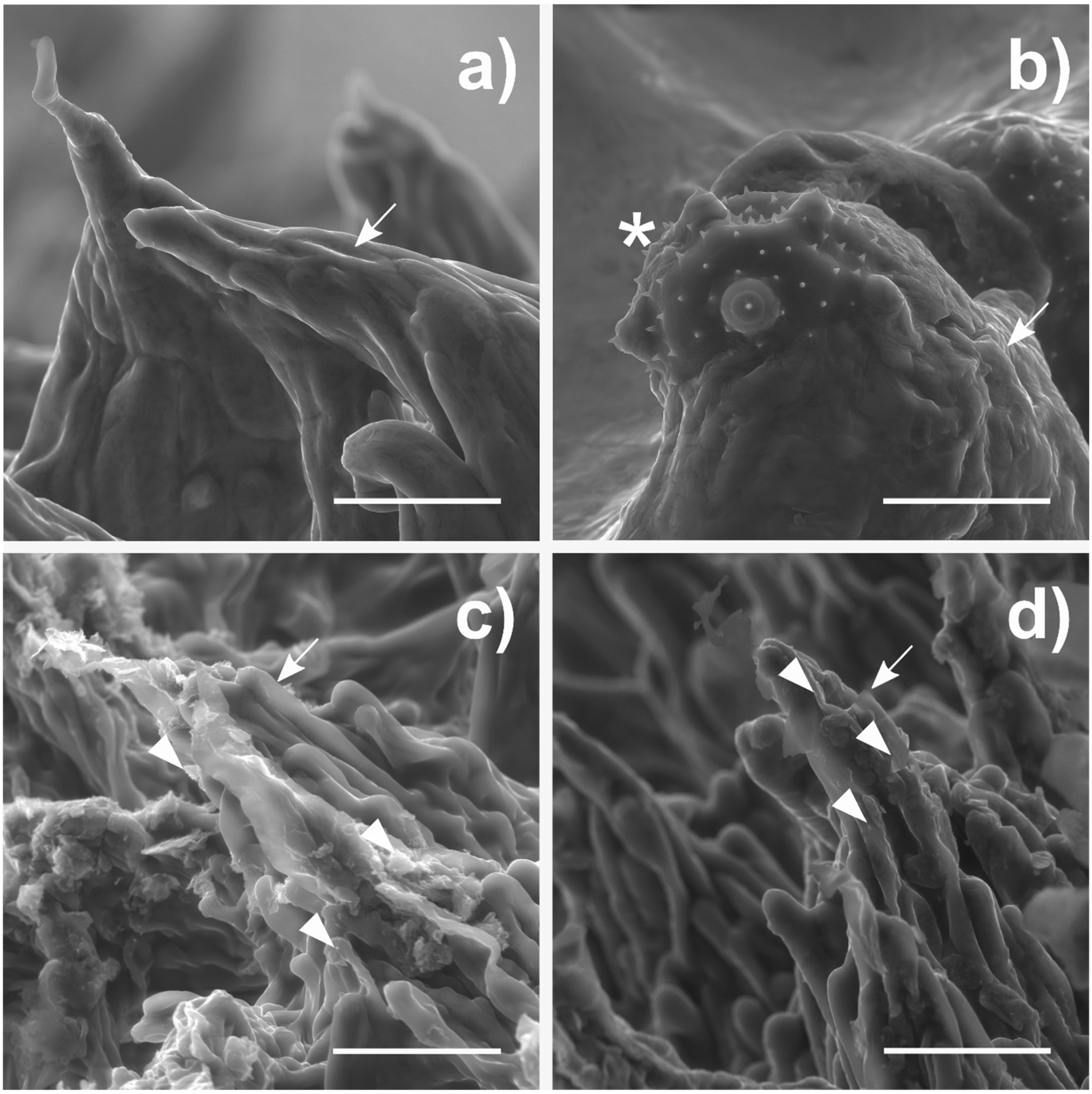

Figure S4. (**a**, **b**) SEM micrographs of stigmatic papillae of *Cucurbita pepo* female flowers treated for three hours without nanomaterials (CTRL); (**c**) with 1 mg of few-layer graphene (FLG); (**d**) with 1 mg of muscovite mica (MICA). Stigmatic papillae are indicated with arrows, germinating pollen grain with asterisk, nanomaterials with arrowheads. Bars = 100 μm.
